# Nontuberculous Mycobacteria: Epidemiologic, Mycobacteriologic, and Clinical Aspects

**DOI:** 10.1155/2015/523697

**Published:** 2015-06-16

**Authors:** Mehdi Mirsaeidi, Parissa Farnia, Ruxana Sadikot, Po-Ren Hsueh, Stefano Aliberti

**Affiliations:** ^1^Division of Pulmonary and Critical Care, University of Illinois, Chicago, USA; ^2^Department of Mycobacteriology, National Research Center of Tuberculosis and Lung Disease, Shahid Beheshti University of Medical Sciences, Tehran, Iran; ^3^Division of Pulmonary and Critical Care, University of Emory, Atlanta, GA, USA; ^4^Divisions of Clinical Microbiology and Infectious Diseases, Departments of Laboratory Medicine and Internal Medicine, National Taiwan University College of Medicine, Taipei, Taiwan; ^5^Department of Health Science, University of Milano-Bicocca, San Gerardo Hospital, Via Pergolesi 33, 20900 Monza, Italy

Nontuberculous mycobacteria (NTM) are ubiquitous organisms that are highly prevalent in the environment. The evidence that these organisms originate in the environment is overwhelming; and there is no significant evidence for animal-to-human or human-to-human transmission. NTM were first recognized as human pathogens in 1950s [1] and since then more than 150 NTM species have been recognized [2]. Humans may be exposed to NTM on a regular basis through showering, bathing, and other activities using water, soil, or aerosols [3]. How, when, why, and in which population NTM infection might occur are largely unknown.

The incidence rate of NTM infections appears to have increased during the last decade globally resulting in a significant mortality and morbidity [4, 5]. Studies have shown a high incidence rate of NTM in elderly population and increasing susceptibility in very elderly people. It has been estimated that NTM incidence will continue to rise due to increasing elderly population by 2050 [6]. Little is known about the diseases caused by the NTM and only limited data are available showing its prevalence and associated factors [7, 8]. Bronchiectasis, chronic lung diseases, pulmonary fibrosis, and decreased lung immunity are some of known risk factors for pulmonary NTM diseases. NTM disease has also gained increasing attention in countries with high incidence of tuberculosis due to risk of misdiagnosis with multidrug resistant tuberculosis (MDR-TB) [9]. A recently published study showed the frequency of NTM misdiagnosis in up to 30% of patients with suspected MDR-TB [10].

The disease caused by NTM forms a broad clinical spectrum from asymptomatic infection to fatal disseminated disease in human. While many of NTM patients have underlying pulmonary diseases, NTM symptoms are undifferentiated from symptoms of patients with chronic pulmonary structural diseases such as bronchiectasis. Therefore, routine screening and having high suspicion for NTM disease are highly recommended.

Diagnosis of infection by NTM is another challenging issue. The diagnosis of NTM with conventional microbiologic methods may be misleading and molecular approaches are advised [11].

Increased knowledge and understanding of this type of mycobacterial disease could improve patient care of affected persons worldwide. We also highly recommend continuing translational studies to develop precision medicine in mycobacterial diseases [12].

In this special issue, a series of original articles as well as expert reviews on NTM are published to stimulate a greater understanding of NTM infections. Suggested areas of research are included: microbiologic and molecular diagnostic tools, drug susceptibility tests, human genetic susceptibility, prevalence and incidence studies, clinical presentations, and clinical trials for antibiotic therapy. We were particularly interested in studies describing new laboratory diagnostic tools for NTM, modalities for clinical characterization of NTM diseases in patients with and without underlying diseases, and measuring outcomes of treatment trials.

In conclusion, the incidence of NTM disease is increasing and needs urgent attention by health policy makers and international societies. Better molecular tools for rapid diagnosis of NTM, understanding of immunopathogenesis, and host pathogen interactions are needed. In the last years, we have seen an explosion in NTM researches. Consequently, we hope to see more publications from basic and traditional research to answer our questions about NTM in coming years.
